# Comorbidity of periodontal disease: two sides of the same coin? An introduction for the clinician

**DOI:** 10.1080/20002297.2017.1332710

**Published:** 2017-06-14

**Authors:** Palle Holmstrup, Christian Damgaard, Ingar Olsen, Björn Klinge, Allan Flyvbjerg, Claus Henrik Nielsen, Peter Riis Hansen

**Affiliations:** ^a^ Section for Periodontology, Department of Odontology, Faculty of Health and Medical Sciences, University of Copenhagen, Copenhagen, Denmark; ^b^ Institute for Inflammation Research, Center for Rheumatology and Spine Diseases, Rigshospitalet, Copenhagen University Hospital, Copenhagen, Denmark; ^c^ Department of Oral Biology, Faculty of Dentistry, University of Oslo, Oslo, Norway; ^d^ Department of Periodontology, Faculty of Odontology, Malmö University, Malmö, Sweden; ^e^ Division of Periodontology, Department of Dental Medicine, Karolinska Institutet, Stockholm, Sweden; ^f^ Steno Diabetes Center Copenhagen, Copenhagen, Denmark; ^g^ Cardiology Department, Herlev and Gentofte Hospital, Hellerup, Denmark

**Keywords:** Periodontitis, periodontal disease, low-grade inflammation, comorbidity, cardiovascular disease, type 2 diabetes, rheumatoid arthritis, osteoporosis, Parkinson’s disease, Alzheimer’s disease, psoriasis, pneumonia

## Abstract

Increasing evidence has suggested an independent association between periodontitis and a range of comorbidities, for example cardiovascular disease, type 2 diabetes, rheumatoid arthritis, osteoporosis, Parkinson’s disease, Alzheimer’s disease, psoriasis, and respiratory infections. Shared inflammatory pathways are likely to contribute to this association, but distinct causal mechanisms remain to be defined. Some of these comorbid conditions may improve by periodontal treatment, and a bidirectional relationship may exist, where, for example, treatment of diabetes can improve periodontal status. The present article presents an overview of the evidence linking periodontitis with selected systemic diseases and calls for increased cooperation between dentists and medical doctors to provide optimal screening, treatment, and prevention of both periodontitis and its comorbidities.

## Introduction

Periodontitis (PDIS) is a common oral disease, the manifestations of which accumulate with increasing age. Often, one gets the impression that PDIS is generally regarded as a natural, almost inevitable physiological consequence of the aging process. It is important to change this outdated perception. The population and the overall health sector should understand that PDIS is an inflammatory disease linked to the individual’s oral microbiota and immune system [1], and that the patients with PDIS, independent of age, benefit from periodontal treatment [2]. As outlined below, a number of other common medical disorders have inflammatory backgrounds too, which may, at least in part, explain their comorbidity with PDIS.

The oral cavity harbors a large amount of bacteria. By using molecular methods, it is now possible to identify precisely and rapidly >700 bacterial species that comprise the oral microbiome, and over the past 15 years, 68% of oral bacterial species in the mouth have been cultured [3]. Of further interest is that the oral microbiome appears to be individualized, implying that it can vary quantitatively and qualitatively between individuals, although there are significant overlaps. Moreover, there is significant variation in the microbiota at different sites of the oral cavity in one person [3,4]. It is also clear that the oral microbiota changes in relation to different diseases such as PDIS, caries, root canal infections, and mucositis [5]. For example, there is an abundance of anaerobic bacteria in the oral cavity, some of which commonly associates with PDIS. These include *Porphyromonas gingivalis*, *Tannerella forsythia*, and *Treponema denticola* (the three members of the ‘red complex’). Moreover, several bacterial species associated with PDIS have been suggested to be involved in the pathogenesis of a number of systemic diseases [6,7].

In the latest decades, there has been considerable scientific interest in the connection of PDIS with a number of medical diseases, several of which also have a high prevalence in the general population. Limited or no solid evidence exists for a direct causal relationship between PDIS and other inflammatory diseases, and documentation in the form of randomized clinical trials is, however, unlikely ever to be achieved. For example, randomized trials of effects of PDIS treatment on the incidence of medical comorbidities, for example cardiovascular disease (CVD) or diabetes mellitus (DM), would require long-term follow-up of an enormous number of patients and be limited by treatment blinding issues and ethical reservations by leaving PDIS untreated in the placebo group. Therefore, health authorities must rely on an assessment of the accumulated plausibility of a causal relationship between PDIS and its comorbidities, which will probably forever remain based on a sum of indirect evidence.

## PDIS and CVD

Atherosclerosis, the major cause of CVD, is an inflammatory disease that develops in the large arteries, and is responsible for ischemic heart disease, stroke, and peripheral artery disease. Atheromatous plaques are usually asymptomatic until they become unstable with plaque rupture/erosion and thrombosis that are associated with increased inflammatory activity in both the arterial wall and systemically in the body [8]. As indicated above, the question of whether the relationship between PDIS and atherothrombosis is causal is difficult to answer definitively. The present review will focus on clinical studies addressing this issue while results of numerous exciting experimental studies supporting the relationship are beyond the scope of the review.

In the recent Swedish ‘PAROKRANK’ study including 805 patients <75 years of age with first-time acute myocardial infarction (AMI) and 805 matched controls without AMI, clinical dental examination and panoramic X-rays were conducted on all participants [9]. PDIS, verified by radiographically rated bone loss, was more common in patients with AMI than it was in controls. There was an increased (+49%) risk of AMI among the PDIS patients. The risk remained significantly increased (+28%) after adjustment for co-variables (smoking, DM, socioeconomic factors). These findings from the largest and most well-conducted case-control study to date emphasize that there can be an independent association between PDIS and AMI [9], which was supported by another recent study from Scandinavia [10].

## Explanatory models

Numerous other population studies, including different ethnicities, have shown a connection between PDIS and CVD, and there is increasing evidence for this connection [11]. This link may be explained by several, not mutually exclusive, mechanisms (see Table 1).

**Table 1.** Explanatory models for association of PDIS and CVD

*Transfer of periodontal bacteria to atheromatous plaques*	In subjects with periodontal inflammation, daily oral activities, including chewing and oral hygiene procedures, may result in transfer of periodontal bacteria from the inflamed pockets to the bloodstream [12]. A following infection of atherosclerotic arterial walls may result in instability of plaques with rupture and thrombus formation [13,14]. Studies have demonstrated the presence of DNA from oral bacteria in atheromatous plaques [15], and some studies using advanced cultivation techniques have also shown viable periodontal bacteria in the plaques [6,16].
*Spillover of cytokines from periodontal tissues to the bloodstream*	Inflammation in the periodontal tissues involves local production of proinflammatory cytokines [17]. Spillover of such cytokines and cytokine producing cells to the bloodstream may enhance inflammation in the atherosclerotic arterial walls and result in unstable plaques. Thus, elevated levels of some proinflammatory cytokines, including interleukin (IL)-6 and tumor necrosis factor alpha, have been found in the bloodstream of patients with PDIS, and PDIS may thus cause systemic low-grade inflammation [18–21] promoting, for example, endothelial dysfunction and development of insulin resistance.
*Systemic production of cytokines*	Increased plasma level of cytokines in response to bacteremia after oral procedures is a well-described phenomenon. For example, increased IL-6 levels were found in the bloodstream as a result of bacteremia after scaling [22], and this cytokine is a known risk marker for CVD, including AMI [23,24].
*Change of lipid metabolism as a result of PDIS*	In patients with PDIS, the lipid balance in the bloodstream, disturbance of which is associated with increased risk of atherosclerotic disease, shows an unfavorable shift with less high-density lipoprotein and more low-density lipoprotein cholesterol [25,26]. In addition, studies have indicated improvement of lipid parameters after periodontal treatment [21].
*Endothelial dysfunction*	PDIS is associated with endothelial dysfunction, which is considered the earliest marker of atherosclerosis [27]. Furthermore, periodontal treatment may improve endothelial function [28].
*Shared genetic risk factors*	Obviously, there may be unknown shared risk factors of importance, including the genetic profile of patients with the two diseases. For example, a shared variant in the IL-1 gene complex could be part of the background for the simultaneous occurrence of both diseases, and recent studies have identified a number of other shared genetic risk factors [29,30]. However, these factors do not appear to explain the observed association fully.

PDIS, periodontitis; CVD, cardiovascular disease; AMI, acute myocardial infarction.

## Significance of periodontal treatment

As indicated above, there are several options for PDIS to affect the development of atherosclerosis and its clinical manifestations. The question is whether periodontal treatment can influence this process. A recent systematic review with meta-analysis concluded that periodontal treatment improves a number of surrogate measures for atherosclerosis, including endothelial dysfunction and lipid parameters, glycated hemoglobin (HbA1c), and biomarkers such as high sensitive C-reactive protein and interleukin (IL)-6, especially among those who already suffer from coronary heart disease and DM [21]. A longitudinal study from the United States has also shown that improvement of the periodontal status with reduced clinical probing depth and diminished subgingival presence of bacteria associated with PDIS among 420 participants resulted in a reduced progression of carotid intima-media thickness (IMT) over 3 years, and the average progression of carotid IMT was inversely correlated with the improvement of periodontal status [31]. According to the authors, the study emphasized the significance of periodontal treatment as a possible preventive health effort. As mentioned above, endothelial dysfunction and carotid IMT are surrogate measures for atherosclerosis. While the importance of periodontal treatment for reduction of clinical cardiovascular endpoints has been suggested in epidemiological studies, for ethical reasons, randomized trials are unlikely to be performed in this area of research.

A comprehensive longitudinal study in Taiwan with an average follow-up period of 7 years was based on a random sample of one million people [32]. It was attended by 10,887 people, who had received dental treatment during the study period. A total of 10,989 age-, sex-, and comorbidity-matched subjects who had not received dental treatment were also included. In the scaling group, a significantly lower incidence of AMI (1.6% vs. 2.2%; *p* < 0.001) and stroke (8.9% vs. 10%; *p* = 0.03) was seen. A multivariate analysis showed that scaling was independently associated with significantly reduced risk of AMI (hazard ratio [HR] = 0.69) and stroke (HR = 0.85). Furthermore, there was a dose-dependent correlation with increased frequency of scaling leading to greater reduction in the risk of AMI and stroke. A weakness of this study was, however, that correction for all known risk factors such as smoking was not performed [32]. In a recent longitudinal study, also from Taiwan, 13,573 patients were treated for mild PDIS in the period 2001–2010, and an equal number of matched patients were treated for severe PDIS [33]. Among the latter patients, those who were >60 years of age had more frequent cardiovascular events, suggesting that the severity of PDIS plays a role in these events.

## Hypertension

Hypertension is associated with PDIS [34,35], and these conditions occur frequently in patients with greater attachment loss [36]. Since hypertension is a treatable risk factor for CVD, it is important to identify patients with hypertension. Therefore, it should be considered whether dentists can contribute to such screening, since patients usually visit dentists more frequently than they visit medical doctors for preventive healthcare measures in the absence of known disease. Sublingual varices are associated with hypertension [37], and this oral manifestation may be used as indicator for screening and referral of patients to their general physician. Moreover, for the dentist, it is also important to know if the patient has hypertension, which may contribute to increased bleeding during oral surgery. In addition, many patients with hypertension are treated with calcium antagonists that occasionally can cause gingival hyperplasia, which again may result in increased progression of PDIS [38]. Obviously, it is up to the dentist to disrupt this potentially vicious circle.

## PDIS and type 2 diabetes

It is well known that there is a relationship between DM and PDIS. As a result of the obesity epidemic, there has been a significant growth in the number of patients with type 2 diabetes (T2D) [39], and it is expected that dentists will receive increasing numbers of such patients for diagnosis and treatment in the future. The relationship is bidirectional in that DM predisposes for PDIS [40], and PDIS can worsen the course of DM, as recently reviewed [41].

## Explanatory models

There are several ways by which PDIS and T2D may interfere with each other. DM can affect the development of PDIS through a change in the oral microbiota, although it is still uncertain whether such a change actually takes place [42]. The main factor for the increased propensity to develop PDIS among diabetics is probably the formation of advanced glycation end products (AGE) by glycation of proteins and lipids [43]. At high blood-sugar levels, characteristic of poorly controlled DM, the formation of AGE is increased, and the receptors for AGE (RAGE) are also upregulated, which leads to increased production of proinflammatory cytokines and increased tissue degradation, including increased bone resorption and decreased bone formation [44,45]. In addition, there are studies suggesting that DM patients display altered function of neutrophils, which play a major role in the pathogenesis of PDIS [46]. It is important to emphasize that well-controlled DM patients are not at increased risk of PDIS. It is thus important for the dentist to have information about blood-sugar control in the individual DM patient. On the other hand, epidemiological studies have linked PDIS to insulin resistance, and PDIS appears to be an independent risk factor for T2D [47,48].

## Undiagnosed diabetes

Not all patients with T2D are aware that they have the disease because the initial symptoms are mild, and probably almost half of these patients are undiagnosed. Because it is critical for prevention of DM complications, including eye disease, kidney disease, neuropathy, and CVD, DM must be diagnosed as early as possible, and from an individual as well as societal and economic perspective, it is very unfortunate if diagnosis is delayed. It is also disadvantageous for dental treatment that the diabetic state is unknown. In the above-mentioned Swedish PAROKRANK study [9], in which patients with a first AMI were compared to controls without ischemic heart disease, glucose metabolism was examined by oral glucose tolerance test, and 9.3% of patients with AMI and 5.2% of the control group had undiagnosed DM. Another recent study revealed that 3.1% of 291 patients without diagnosed T2D who sought dental treatment at the Department of Odontology at the University of Copenhagen had HbA1c above the threshold for T2D, and similarly 27.1% had HbA1c above the threshold for pre-diabetes [49]. Pre-diabetes is a condition where blood-glucose levels are above the normal, but still do not qualify for the T2D diagnosis. This condition, which is a precursor of manifest T2D, is also known as impaired glucose tolerance. Patients with PDIS more frequently had elevated HbA1c than the control group without PDIS did. It is easy and cheap to implement screening for elevated HbA1c, and since many patients visit the dentist more regularly than they do the medical doctor, irrespective of whether they feel healthy, there is a golden opportunity to implement HbA1c screening in selected risk patients in dental clinics, with referral to their general physician in the case of elevated values.

## Significance of periodontal treatment

Studies on the significance of periodontal treatment for the course of T2D often carry considerable methodological limitations, for example missing sufficient confounder control and with incomplete information on the efficacy of the periodontal treatment. However, several meta-analyses have shown that non-surgical periodontal treatment reduces HbA1c levels significantly in the range of 0.31–0.65% [50,51]. Even such small reductions in HbA1c can be clinically important. Thus, a large British study demonstrated that every percentage point decrease in HbA1c may result in as much as 35% reduction of microvascular complications, and an average reduction in HbA1c of 0.2% was related to a 10% lower mortality rate [52]. Therefore, the reduction by 0.31–0.65%, which can be achieved by periodontal treatment, can have a great impact in terms of systemic health and societal economy.

## PDIS and rheumatoid arthritis

Rheumatoid arthritis (RA) is an autoimmune disease affecting 0.5–1% of the population in the Western world. The disease involves inflammation of the joints, with cartilage degradation and joint deformity, swelling, and pain [53]. Like PDIS, RA is a chronic inflammatory condition, which leads to tissue degradation, and an association between the two diseases has been demonstrated, as recently reviewed [54]. Despite a limited number of participants, the available studies suggest that both younger and older patients with RA have an increased predisposition to attachment loss [55–58]. This might argue for establishing periodontal prevention programs as part of routine treatment of patients with RA [55–58].

## Explanatory models

PDIS and RA may associate bidirectionally. Both diseases display elevated circulating and target tissue levels of markers of inflammation and cytokine profiles of ‘tissue degrading’ nature, including increased production of IL-1 and tumor necrosis factor alpha (TNF-α) [55–59]. RA is furthermore characterized by the formation of autoantibodies, including rheumatoid factors recognizing immunoglobulin G (IgG) and antibodies to citrullinated proteins (ACPAs) [60]. The latter are found in approximately three-quarters of RA patients, which also have a characteristic expression of major histocompability complex molecules capable of binding citrullinated peptides [61]. Indeed, the subgroups of RA patients who show immune responses to citrullinated proteins and those who do not are considered by many investigators to be two distinct disease entities. Post-translational conversion of the amino acid arginine to citrulline is catalyzed by enzymes of the peptidylarginine deiminase (PAD) family, and these are considered important in disease progression, at least in ACPA-positive RA [60].

Patients with antibodies against citrullinated proteins more frequently appear to have PDIS than patients with osteoarthritis do [62]. In addition, RA patients more frequently have antibodies against *Porphyromonas gingivalis* than healthy controls do [63]. In search for a mechanistic link between PDIS and RA, special attention has been drawn to *P. gingivalis*, which is the only bacterium with capacity to produce a PAD (PPAD). Like the corresponding human enzyme, PPAD is capable of converting arginine to citrulline [64]. In theory, PPAD may therefore convert harmless host proteins into citrullinated autoantigens that become the target for autoantibodies and pathogenic T cells that drive RA. Smoking, which increases the risk of PDIS, is also the strongest life-style factor linked to the development of RA. Smoking is also believed to promote the secretion of PAD from leukocytes in the lungs and thus initiate citrullination [65].

## Significance of periodontal treatment

Several studies have evaluated the effect of periodontal treatment on RA and biomarkers of the disease [58]. The available studies, however, are small and with limited follow-up, but they suggest that non-surgical periodontal treatment may reduce clinical symptoms and biomarkers of active RA. Major intervention studies in RA patients with PDIS are needed in order to draw firm conclusions on this matter.

## PDIS and osteoporosis

A possible association between PDIS and osteoporosis was described already in 1968 [66]. Osteoporosis is a systemic skeletal disease characterized by reduced bone density. Clinically, osteoporosis is divided into (1) an idiopathic form that appears early in life and affects men and women with equal frequency, and (2) an involutional form, which is subdivided into two types, the first of which includes postmenopausal women, and the other being age-related and including both elder men and women [67]. The most prevalent form is postmenopausal osteoporosis, the possible association of which with PDIS has been examined in several clinical studies. The majority of these studies have been cross-sectional and with few participants, all postmenopausal women [68–75]. A recent cross-sectional study from Taiwan including 35,127 osteoporosis patients and 50,498 healthy controls showed that PDIS was associated with an increased risk of osteoporosis (odds ratio [OR] = 1.29) after adjustment for sex, age, and comorbidity, and that the risk increased with increased degree of periodontal inflammation [76]. Furthermore, osteoporosis was associated with a sixfold increased risk of concurrent PDIS. These results are supported by a second cross-sectional study from South Korea, which showed a positive correlation between PDIS and osteoporosis (OR = 1.21) after adjustment for age [77]. However, longitudinal studies are missing to substantiate a causal relationship between PDIS and osteoporosis.

## Explanatory models

Various systemic risk factors, such as genetics, age, sex, vitamin D deficiency, medical hormone therapy, diet, smoking, obesity, and physical activity, affects the development of osteoporosis [78,79], but several of these are also risk factors for PDIS [80]. Bone density changes throughout life, but after the menopause a decrease in estrogen production occurs, which seems to be associated with an increased risk of osteoporosis. Decreased bone density in the jaw bone in subjects with osteoporosis is obviously compatible with this condition, leading to attachment loss in individuals with PDIS [72,73,81,82]. Besides being associated with decreased bone density, estrogen deficiency also affects the other periodontal tissues and the immune response against the periodontal biofilm in a proinflammatory direction [81].

## Significance of periodontal treatment

As yet, no studies have evaluated the effect of periodontal treatment on osteoporosis. Furthermore, it remains unclear whether bisphosphonate treatment of postmenopausal patients with osteoporosis worsens or improves periodontal parameters. One study, however, showed that bisphosphonate therapy did not reduce alveolar bone loss in osteoporosis patients with PDIS [82].

## PDIS and Alzheimer’s disease

Alzheimer’s disease (AD) is a neurodegenerative disease and the most common example of a group of diseases causing dementia. It is a progressive disease, with susceptibility genes working with little-understood environmental and behavioral influences [83]. AD is characterized by atrophy and neuronal death, especially in the hippocampal region of the brain [84]. There are two main categories of AD: the familial, early-onset form that targets individuals <65 years of age and accounts for about 2% of all cases of AD; and the late-onset form of AD that affects older (>65 years) subjects and accounts for approximately 98% of the cases. Late-onset AD has several genetic susceptibility traits. Among these, the apolipoprotein *APOE* ɛ4 allele is considered to be the most important [85]. The disease is already a great economic burden for society, and there is currently no treatment. Late AD probably has several causes, while a genetic component is more essential for the early form. Characteristically, late AD includes inflammatory changes in the brain, which may be initiated by local or systemic infection [86].

Among the microorganisms most frequently associated with AD are bacteria such as spirochetes, *P. gingivalis*, *Prevotella*, fusobacteria, *Actinomyces*, and *Chlamydophila pneumoniae*. Also, herpes virus (Epstein–Barr virus and cytomegalovirus) and yeasts of the genus *Candida* have been connected with AD [83]. With the exception of *C. pneumoniae*, all these microorganisms can be present in the periodontal pockets.

There is emerging evidence of a link between PDIS and AD. The relationship has been shown in cross-sectional and longitudinal studies by examining the association of AD with clinical signs of PDIS and circulating levels of antibodies against bacteria associated with PDIS, respectively [84].

## Explanatory models

### Association of AD with periodontal bacteria

In addition to the red complex bacteria, *Fusobacterium nucleatum* and *Prevotella intermedia*, both known to be associated with PDIS, also associate with AD. Indeed, in the National Health and Nutrition Examination Survey (NHANES), antibody levels to these organisms were significantly increased in serum from patients with AD compared to controls [87]. This result was significant after controlling for each subject’s age, Mini-Mental State Examination score, and *APOE*ԑ4 allele status. Unexpectedly, Noble et al. found that a high (>640 ng/mL) anti-*Actinomyces naeslundii* titer was present in 10% of subjects with increased risk of AD, suggesting that AD pathogenesis may involve a spectrum of bacteria [88].

In 14 studies, oral spirochetes that are neurotrophic were demonstrated in the brain of AD patients. Seven different spirochetes were identified in 14/16 AD brains [89,90]. Spirochetes induced biological and pathological characteristics of AD (plaque accumulations of beta-amyloid and neurofibrillary tangles) after exposure of neuronal and glial cells in organ cultures. Lipopolysaccharide (LPS) from *P. gingivalis* was also detected in human brains with AD but not in control brains [91]. In a study based on 2,355 people >60 years of age, a positive correlation between PDIS and cognitive impairment was found, and a negative correlation was observed between antibody titers to *P. gingivalis* and scores in cognitive tests [87,92].

The original inflammatory hypothesis of AD suggested that AD hallmark proteins, for example beta-amyloid, were the main contributors to central nervous system inflammation. This hypothesis has been expanded to include involvement of infections, and life-style, genetic, and environmental factors in AD pathogenesis. PDIS is a prototypical oral condition that encompasses all these factors, including pathogenic bacteria [93].

### Microorganisms and inflammatory mediators may reach the brain

Oral microorganisms and inflammatory mediators can be transported from inflamed periodontal tissues to the brain via the bloodstream. An increased amount of cytokines, particularly the macrophage-secreted TNF-α, has been detected in the plasma of AD subjects [94]. Also, elderly people harbored a higher titer of circulating IgG against several periodontal pathogens [87]. Cestari et al. [95] found an association between circulating IL-6 and TNF-α levels in patients with AD and PDIS, implicating these proinflammatory cytokines in the overlapping pathogenic mechanisms between oral infections and AD [95].

As mentioned above, daily episodes of bacteremia follow from dental procedures, including toothbrushing and flossing, and from chewing, particularly in patients with PDIS. The bacteria involved can disseminate into the brain by closely related anatomical pathways, that is, trigeminal and olfactorial nerves [93]. The long-term effect of inflammatory mediators and pathogens and/or their virulence factors reaching the brain may over time prime the brain’s own microglia in individuals having inherent susceptibility traits. According to Singhrao et al., such susceptibilities could contribute to inadequate neutralization of invading agents reaching the brain and result in loss of cytoarchitectural integrity and vital neurons with subsequent deterioration of cognitive function [93].

#### Blood–brain barrier

The permeability of the blood–brain barrier (BBB) increases with age. Prolonged exposure to high concentrations of TNF-α tends to weaken the protective role of the BBB, making it more permeable to bacteria or endotoxins [96]. The *APOE ԑ4* gene is also associated with increased BBB permeability, allowing microorganisms, their products, and inflammatory mediators such as TNF-α to penetrate into the brain [97]. These microorganisms and substances can also pass through circumventricular tissues and perivascular spaces of the brain because these regions lack a BBB [98]. The olfactory nerve and the trigeminal nerve also circumvent the BBB [99]. Indeed, olfactory cells may act as Trojan horses by which microorganisms can reach the brain [100].

#### Bacteria in the brain

Biofilm has been demonstrated in the brain of AD patients and was probably created by dental and Lyme spirochetes with accompanying local tissue activation of the innate immune system [101]. Riviere et al. also demonstrated the presence of seven different *Treponema* species in 14/16 specimens from AD brains [102]. Microorganisms and their toxic products as well as microbial DNA have been reported in the brain tissue of AD patients and animal models [83]. Spirochetes induce a latent and slowly progressive infection by circumventing host defense, and are able to induce beta-amyloid plaque formation in the brain [87,92]. Periodontal bacteria, especially *T. denticola*, may contribute to AD pathology using a range of inflammatory mechanisms by which neurons would be attacked. This occurs despite the fact that these bacteria inhibit inflammasome activity [103,104]. Spirochetes possibly promote their own survival and proliferation by blocking the complement cascade [105]. Moreover, *P. gingivalis* has LPS with various lipid A structures and is capable of modifying the latter components, which may provide the bacteria with the capacity to disguise itself from recognition by the immune system via TLR4 [106].

### Genetics, environmental factors, nutrition, and other factors

A very important risk factor for AD is the *APOE ԑ4* gene, which is associated with susceptibility for infections and increases the expression of inflammatory mediators [107]. In total, 20 different genetic loci have been estimated to increase the susceptibility to AD, including *APOE ԑ4*. These include the genes for IL-1β and TNF-α, which are also linked to the development of PDIS [108]. The pathogenesis of AD probably includes an interaction between genes, microorganisms/toxins, and environmental factors. Inadequate nutrient intake is common in the elderly and in people with dementia, and this can contribute to gradual loss of nerve synapses. In addition, neglect of or an inability to maintain oral hygiene in the elderly promotes inflammation in the periodontium, which may favor the transport of microorganisms and their products, as well as inflammatory mediators, to the brain. Loss of teeth, which is often the result of PDIS, has been connected to a poor memory [109].

## PDIS and Parkinson’s disease

Parkinson’s disease is another chronic neurodegenerative disease that results in selective loss of dopaminergic neurons in the substantia nigra of the brain. During the progression of Parkinson’s disease, there is a gradual degeneration of the nigrostriatal compounds, leading to cognitive, motor, and psychiatric symptoms. There is still no solid evidence that PDIS influences the pathogenesis of Parkinson’s disease [110]. However, there are studies indicating that PDIS is more common in patients with Parkinson’s disease, although large longitudinal studies and randomized case-control or case-cohort studies are lacking to substantiate this association [111,112].

## Explanatory models

Parkinson’s disease causes motor disability, which complicates the provision of simple daily oral procedures such as brushing the tooth, which will inevitably lead to the accumulation of plaque. In addition, the cognitive changes in patients with Parkinson’s disease may have an impact on the quality and frequency of the home dental-care habits (as well as the dentists’ willingness to perform periodontal treatment), which contributes to increased plaque accumulation and risk of PDIS. A number of studies also indicate that systemic low-grade inflammation induced by PDIS [17,19,22] contributes to neural dysfunction at early stages of Parkinson’s disease [110]. Much evidence suggests that the pathogenesis of Parkinson’s disease has an inflammatory component, for example elevated plasma IL-6 appears to increase the risk of the disease [113,114]. There are no published studies on the effect of periodontal treatment of patients with Parkinson’s disease.

## PDIS and psoriasis

Psoriasis is a chronic inflammatory disease with a prevalence of up to 8.5% of the population in the Nordic countries. The disease is also characterized by extensive comorbidity in the form of, for example, CVD and T2D, probably on the basis of shared inflammatory mechanisms [115–117]. An association between psoriasis and chronic PDIS has been shown, and increased concentrations of proinflammatory cytokines such as TNF-α and IL-1β have been found in saliva from patients with psoriasis [118–120]. The results from a large epidemiological study from Taiwan also suggest that intensive treatment of chronic PDIS can reduce the risk of psoriasis [121]. Activated T-helper (Th)-17 cells producing IL-17 are key pathogenic players in psoriasis, and bacterial infection, including infection with *P. gingivalis*, can promote the polarization of naïve T-helper cells into Th-17 cells. Also, activated Th-17 cells have been found in periodontal lesions, and increased IL-17 levels have been demonstrated in crevicular fluid from patients with MP [122–124].

## Lung diseases and oral hygiene

Colonization of the oral cavity with respiratory pathogens related to a lack of oral hygiene and PDIS can be linked to the development of pneumonia. There is strong scientific evidence from randomized clinical trials that interventions aimed at improving oral hygiene may prevent pneumonia and reduce pneumonia-related deaths, particularly in elderly care-dependent patients [125]. A Norwegian study also showed that chronic PDIS occurs more frequently in patients with severe chronic obstructive pulmonary disease, even after adjusting for risk factors such as age, smoking, obesity, corticosteroid use, and decreased bone density [126].

## Conclusion

There are numerous studies showing a correlation between PDIS and a variety of medical disorders. This is not surprising, since the mouth, of course, is part of the human body. These medical conditions are particularly frequent in the elderly population. Important for the dental clinician, there is evidence to suggest that periodontal treatment may have beneficial effects on some of these conditions (Table 2). It is obvious that cooperation between medical doctors and dentists should be strengthened, and a major prerequisite for this is increased awareness and knowledge about the disease connections mentioned in this article. Importantly, low-grade inflammation is considered to be one of the most prominent mechanistic links between PDIS and its medical comorbidities.

**Table 2.** Medical disorders with studies indicating that periodontal treatment has beneficial effect on course of disease or surrogate measures of disease

Disorder	Reference
CVD	[21,31–33]
T2D	[50,51]
RA	[58]

T2D, type 2 diabetes; RA, rheumatoid arthritis.

A considerable amount of knowledge has accumulated about the link between PDIS and CVD, with growing evidence for a causal relationship. There are plausible mechanistic data (including experimental results that are not discussed here), and it appears that periodontal treatment may reduce the risk of atherosclerotic disease.

There is also extensive evidence that poor blood-sugar control in patients with T2D leads to an increased risk of PDIS with increased severity and extension, and that PDIS may lead to increased risk of elevated HbA1c and T2D. The growing prevalence of T2D in the populations will probably result in increased development of PDIS, which in turn can aggravate the course of T2D. Thus, the two diseases have a bidirectional relationship, presumably due to shared immunological reactions. Clinical studies also indicate that non-surgical periodontal treatment can improve metabolic control, which may reduce the development of diabetic complications. There are ample reasons for establishing systematic examination, prevention, and therapy programs for PDIS in diabetic patients.

Evidence suggests that there is also a bidirectional link between PDIS and RA, with increased risk for PDIS in patients with RA, and non-surgical periodontal treatment may reduce clinical symptoms and biomarkers of active RA. The increased propensity for attachment loss in patients with RA can also speak for the establishment of a periodontal prevention program as part of routine treatment of these patients. Moreover, studies of patients with osteoporosis suggest that there is an increased propensity to develop attachment loss.

PDIS is also associated with certain neurological disorders. Parkinson’s disease involves motor impairment and cognitive changes, which may entail deterioration of home dental-care habits. Moreover, low-grade inflammation, for example as a result of PDIS, may contribute to neurological dysfunction in the early stages of Parkinson’s disease. AD has a complex and multifactorial etiology, and periodontal infection may be one of several risk factors for AD. Thus, the presence of periodontal bacteria and their products have been found in the brain of AD patients. Infection can occur decades before AD becomes apparent. Improved oral hygiene can be an important prophylactic measure, but unfortunately can be challenging because AD patients are not always cooperative.

Psoriasis is characterized by widespread comorbidity in the form of, for example, CVD, diabetes, and PDIS, which probably also has a background in shared inflammatory mechanisms. Finally, there is scientific evidence that better oral health has a positive effect in the prevention of pneumonia, especially in elderly care-dependent patients.
